# Exploring Mental Health Issues and Priorities in Indonesia Through Qualitative Expert Consensus

**DOI:** 10.2174/0117450179331951241022175443

**Published:** 2024-11-07

**Authors:** Ray Wagiu Basrowi, Tjhin Wiguna, Kristin Samah, Nila Djuwita F Moeloek, Mudji Soetrisno, Semiarto Aji Purwanto, Maria Ekowati, Adriana Elisabeth, Andre Rahadian, Bacelius Ruru, Bunga Pelangi

**Affiliations:** 1 Caucus of Indonesian Mental Health Care Community (Kaukus Masyarakat Peduli Kesehatan Jiwa), Jakarta, Indonesia; 2 Occupational Medicine Study Program, Department of Community Medicine, Faculty of Medicine, Universitas Indonesia, Jakarta, Indonesia; 3 Health Collaborative Center (HCC), Jakarta, Indonesia; 4 Department of Psychiatry, Faculty of Medicine Universitas Indonesia - Dr. Cipto Mangunkusumo General Hospital, Jakarta, Indonesia; 5 Indonesian Women Empowerment Group (Wanita Indonesia Keren), Jakarta, Indonesia; 6 Indonesian Ophthalmologist Association, PERDAMI, Jakarta, Indonesia; 7 Sekolah Tinggi Filsafat Driyarkara, Jakarta, Indonesia; 8 Faculty of Social and Political Sciences, Universitas Indonesia, Jakarta, Indonesia; 9 Dentons HPRP Law Firm, Jakarta, Indonesia; 10 Podomoro University, Jakarta, Indonesia

**Keywords:** Mental health, Indonesia, Community caucus caring for mental health, Expert consensus, Multi-pronged recommendations, Disorder

## Abstract

**Background:**

Mental health well-being is a fundamental human right. However, mental health awareness is not yet considered as a main priority for the government and public in Indonesia. Thus, there is an urgent need for Indonesians to fully comprehend the importance of raising mental health awareness.

**Methods:**

A discussion among 45 experts in September until October 2023 was conducted by the Community Caucus Caring for Mental Health to raise an awareness of the importance of mental health in Indonesia. The results were reported as an expert consensus.

**Results:**

The discussion acknowledged the urgency of managing mental health issue along with its five key drivers and three essences. It identified nine specific themes of mental health issues in Indonesia by focusing on four high-risk population groups. The consensus proposed multi-pronged recommendations, *i.e*., developing personalized mental health-related campaigns and movements, conducting mental health-relevant studies at clinical and community settings, incorporating of mental health awareness in teaching curriculum and family’s discussion, innovating technology for screening and diagnosing mental health issues, as well as an providing mental health first-aid with wide access to all population.

**Conclusion:**

The expert consensus concluded that Indonesians must start to prioritize mental health awareness and to provide sufficient resources to screen, diagnose and treat individuals with mental health disorders. The expert consensus identified nine specific themes of mental health issues in Indonesia and subsequently proposed multi-pronged recommendations with an aim to improve mental health awareness in Indonesia.

## 1 INTRODUCTION

As mentioned by the WHO, mental health is an integral component of health and well-being, underlying the capacities of individuals and societies to make decisions, build relationships and shape the world that we reside in [1]. It plays a vital role in everyone's life by influencing thought processes, emotions and actions. Possessing good mental health implies the ability to connect, function, cope and thrive in various aspects of life [2]. Thus, mental health well-being is a fundamental human right as there is no health without mental health [1, 2]. Disruptions in mental health conditions and a lack of access to appropriate support can disturb human well-being. On a global scale, an estimated 1 in 8 people (approximately 970 million individuals) live with mental disorders, with anxiety and depression being the most common. Of note, 82% of them reside in developing countries [3]. However, these conditions are often inadequately addressed, as 71% of individuals with psychosis do not receive mental health services. From a resource perspective, only 2% of the health budget is allocated to mental health [3]. Yet, mental health issues and disorders have economic consequences, with the loss of productivity significantly exceeding direct medical treatment costs. The economic impact is projected to reach 6 trillion USD by 2030, which even surpasses an estimate for combined losses from cancer, diabetes, and chronic respiratory diseases. Furthermore, low- and middle-income countries are expected to bear 35% of the cost of mental health conditions [3].

According to The Lancet Commission on Global Mental Health, mental health cases are estimated to incur costs of up to 16 trillion USD from 2011 to 2030, including healthcare facility and medication expenses for patients, therapy, and other expenses, *e.g*., productivity loss, declining social well-being, as well as education and legal matters [4]. Growing evidence suggests that addressing and transforming the mental health agenda are not only about ensuring access to quality services and treatment but also require greater attention and investment in addressing various social and economic issues that shape community mental health. Nations worldwide have committed to addressing these issues by working to achieve the Sustainable Development Goals (SDGs) by 2030 [5]. The relationship between mental health and development goals is complex, often representing a two-way connection. Progress in achieving SDGs has the potential to enhance and protect mental health. Simultaneously, improving overall health status (including mental health) is crucial for fulfilling global SDG commitments [4]. This underscores the significant contribution of mental health to human development efforts. If mental health disorders/issues exist, they can impact a country's achievement of fundamental rights and well-being conditions.

The burden of mental disorders encompasses the entirety of the life journey, ranging from developmental and behavioral disorders in childhood, adolescence, adulthood, and old age. Overall, the heaviest burden occurred during early adulthood, which was dominated by depression and anxiety disorders [6, 7]. Of all mental disorders, a significant portion manifests as years lived with disability (YLDs) rather than premature deaths or years of life lost (YLLs) [6]. Global Burden of Diseases, Injuries, and Risk Factors Study (GBD) 2019 reported that mental disorders were among the top ten leading causes of burden worldwide, with no evidence of global reduction in the burden since 1990 [6]. For the aggregate of mental disorders, Australasia, Latin America, and North America had the highest prevalence worldwide [6]. In the region of Southeast Asia, mental disorders were the second highest cause of YLDs in 2019, *i.e*., 1,350.67 YLDs per 100,000 [3]. Of note, global estimates in 2017 indicated that the World Health Organization (WHO) Southeast Asia Region accounted for around 27% and 23% of all cases of depression and anxiety, respectively [8, 9].

In Indonesia, mental disorders were also the second highest cause of YLDs in 2019, *i.e*., 1,304.36 YLDs per 100,000, suggesting the dire state of mental health awareness and management in the nation [3]. Based on data from the 2018 Indonesia Basic Health Research, the prevalence of emotional mental disorders increased from 6% in 2013 to 9.8% in 2018, which affected over 19 million people. Depression rates reached 6.1%, with only 9% of affected individuals receiving medication or medical treatment [10]. Of note, the prevalence of depression was 5.1% in adolescents and 5.6% in young adults in Indonesia [10, 11]. In 2023, it was reported that the prevalence of people with mental disorders was approximately 20% of the Indonesian population [12]. Thus, Indonesia will need to spend 21.9% of its total GDP from 2012 to 2030 just to address mental health disorders, reinforcing the fact that mental health disorders are the second-largest contributor of non-communicable diseases in Indonesia, just below cardiovascular diseases [13]. Taken together, unmanaged mental health disorders would impact and worsen the overall health status as well as the social and economic status of Indonesia.

Considering the high urgency of managing mental health issues in Indonesia, the Community Caucus Caring for Mental Health was established on November 14, 2023 [14]. The Caucus is a non-profit, community-initiated forum for Indonesians who have interest in co-building and co-raising an awareness of the importance of mental health in Indonesia. The Caucus believes there is an urgent need to set a Mental Health Care Movement in Indonesia, based on joint efforts among communities to advocate, educate, and demonstrate the importance of prevention and mitigation. The Mental Health Care Movement is a community-based initiative aiming to raise awareness and attention among all citizens about the importance of mental health [15]. It advocates for individuals to speak out about the risks, symptoms, or early signs of mental health disorders without feeling shame, fear of stigmatization, and especially without the fear of discrimination. The movement emphasizes that physical health is inseparable from mental health. To initiate this movement, the Community Caucus Caring for Mental Health recently conducted an expert discussion on mental health conditions in Indonesia. The discussion aimed to identify various aspects, including the urgency of mental health, the prioritized issues associated with mental health, as well as the recommended resolution to manage mental health issues. The discussion’s results were proposed as an expert consensus to raise mental health awareness and to determine prioritized mental health issues in Indonesia.

## 2 MATERIALS AND METHODS

### 2.1 Narrative Review

The consensus was constructed based on a narrative review followed by a discussion among invited 45 experts from September until October 2023. The review process analyzed various literature studies, research findings, and reports on the mental health situation globally and in Indonesia. Data analysis was conducted using a content approach by categorizing data based on the priority issues. Relevant studies and policy papers were used as references in this study as well. The review was disseminated to all experts before the meeting.

### 2.2 Expert Consensus based on Convergence Point among Axes

In this step, opinions and views of experts were gathered following the Delphi process approach, *i.e*., (1) expert judgment and (2) expert confirmation [16, 17]. In the expert judgment stage, the experts were asked to participate in filling out the expert response instrument based on Google Forms. All information was interpreted as preliminary expert consensus data. Subsequently, an expert confirmation stage was conducted through an online focus group discussion. This process involved 45 experts to validate and draw conclusions based on the preliminary expert consensus data. Data and information were analyzed using coding and data categorization according to a content analysis approach. Key determinants of challenges, opportunities, priorities, etio- logical indicators, and impact indicators (including spontaneous response & cumulative percentage indicators) were discussed.

### 2.3 Analytical Procedures

The survey results were processed using a descriptive-analytical approach through a mixed-method approach incorporating both quantitative and qualitative methods. Quantitative data were analyzed using the frequency data formula in Microsoft Excel 365 (Microsoft Corporation, Washington) to examine the components of frequency data. This provided numerical insights into the prevalence and distribution of responses. Descriptive data analysis was conducted with a content approach using the NVIVO application (QSR International, Massachusetts). Quali- tative data were processed and displayed using features, such as word clouds. This created a visual representation of the most mentioned themes and concepts within the qualitative responses. Quantitative and qualitative data were synchronized to analyze expert convergence results, enabling a comprehensive understanding of the data. The integration of both quantitative and qualitative approaches helped to complement and deepen the information obtained from the survey responses.

## 3 RESULTS

### 3.1 Challenges of the Mental Health Situation in Indonesia

Forty-nine experts were contacted, out of which 45 responses were obtained (*i.e*., the response rate was 91%). Among those 45 respondents, 41 were affiliated with certain organizations. The respondents were distributed across 11 provinces of Indonesia (of a total of 38 provinces). While sixty-nine percent of the respondents were female, 29% were male and 2% were not willing to be identified. Those experts were categorized into 8 expertise areas, *i.e*., 31% were academicians, 22% were psychologists and psychiatrists, 11% were other medical specialists, 11% were public health practitioners, 9% were members of civil society organizations, 7% were social anthropologists/cultural experts, 4% were media workers and the remaining 5% were workers in other private sector/industry.

In Indonesia, mental health awareness aligns with national development aspirations. As outlined in the 2023 Law No. 17, mental health is a condition where an individual can develop physically, mentally, spiritually, and socially, in which good mental health well-being enables self-awareness, stress management, productive work, and substantial contributions of each individual to the community. This signifies that mental health is a crucial aspect that every individual should possess within the societal framework. However, mental health issues are not properly managed yet in Indonesia. For example, according to the 2022 Indonesia-National Adolescent Mental Health Survey on adolescents aged 10–17 years, poor mental health was a widespread issue among adolescents [7, 18]. The survey reported that 5.1% of adolescents suffer from depression and 9.8% experience emotional and mental disorders. In addition, although happiness plays a vital role in mental health well-being, Indonesia only ranked 84th out of 137 countries in the 2023 World Happiness Report, even below Hong Kong and Albania [19]. Furthermore, the ongoing shortage of specialized mental health professionals in Indonesia contributes to the lack of proper identification and treatment of mental health subjects. Indonesia has a ratio of 0.3 psychiatrists per 100,000 people, which is significantly lower than the global average [20]. Considering these findings, Indonesia is presumed to face a high risk of mental health issues.

On the other hand, the mental health law (2014) was already implemented to address the country's mental health challenges, but its implementation has faced several issues such as slow implementation with significant gaps in enforcement, and resources (shortage of mental health professionals, practical guideline and clarity in integrating mental health services with other services and Only 2% of the health budget was allocated to mental health, with 66.1% directed to mental hospital) [21,22].

The urgency status of the mental health situation in Indonesia was indeed perceived as extremely high by the consensus, in which eighty-two percent of the respondents/experts in Community Caucus Caring for Mental Health, stated that mental health issues were extremely important. This indicated a broad agreement among respondents that mental health issues in Indonesia were a significant concern, which required immediate attention and extensive research. Based on the collected responses, five key drivers that drove the urgency of the mental health issue in Indonesia were identified, *i.e*., (i) multi-sectoral impacts; (ii) serious issues among children, adolescents, and individuals during their productive years; (iii) inadequate information and information distribution; (iv) not prioritized in Indonesia despite a global issue; and (v) root causes encompass economy, social and cultural factors. First, the issue of mental health in Indonesia was multi-sectoral, which stemmed from an integral role of mental health within comprehensive health. Mental health does not only influence productivity but also determines the quality of life and future achievements. The World Health Organization conceptualizes mental health as a state of well-being linked to an individual's ability to act, cope with life stresses, work effectively, and contribute adequately to his or her community. Consequently, an individual's mental health significantly affects various aspects of his or her life, including physical health, employment status, personal abilities, and contributions to society. This strongly emphasized the urgency of addressing mental health issues, as the impact on each of these sectors could lead to harmful and significant changes in an individual's life.

Second, it was acknowledged that the issue of mental health was more prominent and serious among children, adolescents, and individuals in their productive years. This hypothesis was supported by both data and expert consensus, revealing a significant increase in mental health problems during adolescence and productive years in Indonesia, particularly among the workforce population. Similar trends have been observed worldwide, as evidenced by various research findings. A systematic review analyzing child and adolescent mental health problems across multiple countries in the 21st century found that adolescents often began experiencing internalizing symptoms, such as sadness, anxiety, and loneliness [23]. Those mental issues subsequently impacted their academic or working productivity. It was reported that American adolescents typically experienced mental disorders with onset as early as the age of 6 for anxiety, age of 11 for behavioral disorders, and age of 15 for substance-use disorders [24]. As adolescence becomes a vulnerable gateway into adulthood, this becomes a very urgent key driver of mental health issues in Indonesia and worldwide.

Third, lack of awareness and dissemination of inaccurate information regarding mental health issues substantially affected mental health management in Indonesia. This issue mostly stemmed from possible stigma and discrimination within the social and economic layers of Indonesian society. For example, a practice of shackling or “pasung” as a community response to manage individuals with mental health issues is frequently encountered across Indonesia [25, 26]. It still occurs due to stigma and discrimination against individuals with mental health issues in Indonesia. An inverse correlation between the level of knowledge and the level of mental health stigma was observed, suggesting that a lack of knowledge or dissemination of inaccurate information on mental health leads to stigma and discrimination, further exacerbating the mental health issues [25, 26].

Fourth, the mental health issue in Indonesia was not prioritized to the highest level yet. This key driver has indeed been reiterated in various published studies on mental health issues in Indonesia. The lack of prioritization for mental health issues in Indonesia was partially attributed to inadequate legislation to address the problem and insufficiency of the existing legislation in providing care for people and protecting their human rights [27]. The treatment gaps occur due to inadequate skills of health workers in handling mental health disorders and insufficient funding for mental health treatment. As an alternate solution, a co-produced mental health public engagement festival could be an acceptable way to increase awareness of mental health in Indonesian populations, this includes the potential role of midwives as the largest population of health workers in Indonesia [28, 29]. Fifth, the causes and impacts of mental health issues in Indonesia were closely related to economic, social, and cultural factors. As mentioned previously, these issues ranged from treatment gaps and a lack of health workers with the required skills in addressing mental health problems (economic factors) to stigma and discrimination as well as a lack of mental health literacy among the population (social and cultural factors).

All key drivers were substantially influenced by three interconnected essences in Indonesia. The first essence was the high level of stigma and discrimination associated with mental health disorders. This existed across all socioeconomic layers of society as well as across various cultures and demographics. The second essence was a lack of awareness and familiarity in handling mental health issues in specific locations, such as workplaces and educational centers. This contrasted with conditions in developed countries. The third essence was the phenomena of self-diagnosis that led to inappropriate or often delayed treatment. This was partially contributed by a lack of mental health awareness among community members.

### 3.2 Mental Health Priorities in Indonesia

As shown in Fig. (**1**), there were six major mental health priorities in Indonesia by focusing on four high-risk population groups, *i.e*., (i) adolescents with major emphasis on psychological intimidation and sexual violence in family and/or school (at 75.6%); (ii) school-age children with main focus on the learning environment and teaching system (at 51.5%); (iii) workforce population with a consensus on the working environment (at 49.3%); (iv) growth and development of infants and toddlers with a focus on family care (at 33.3%); (v) prenatal and postnatal periods (at 22.2%); and (vi) older adults/elderlies/retired individuals with a focus on being neglected (at 11.8%).

The consensus also identified several minor mental health priorities in Indonesia, including issues on technology dependency (at 5.7%); issues on the broader community/environment (at 4%); issues on minority groups related to gender/sex orientation (at 3.3 political/vertical/horizontal conflicts (at 2.3%); organic madness/schizophrenia (at 1.8%); specific issues within military and religious organizations (at 1.7%); issues related to ethnicity/race/tribe (at 1.3%); mental health issues related to Indonesian traditional beliefs/ancient animism (at 1.3%); issues on post-disaster (at 1.3%); issues due to neglecting of remote/Indigenous areas (at 1.3%); terrorism (at 0.3%); and climate change (at 0.3%).

The consensus acknowledged that there are four groups at high risk of suffering from various mental health issues in Indonesia, including infants and toddlers who go through the first 1,000 days of life (“Mental Health in the First 1,000 Days of Life”), adolescents (“Adolescent Mental Health”), workforce (“Workforce Mental Health”), as well as older adults (“Mental Health among Elderlies”). Of each group, one or two mental health priorities were identified as depicted within ovals.

### 3.3 Specific Mental Health Issues in Indonesia

Based on the six major mental health priorities, the consensus developed nine broad themes, encompassing 27 mental health dimensions of mental health issues (Table **1**). The themes of mental health issues included (A) issues of pregnancy and early parenting (comprising dimensions of (i) prenatal stress and (ii) baby blues syndrome); (B) issues of childhood (covering dimensions of (iii) children with neurodevelopmental disorders, (iv) emotional and behavioral problems, (v) impact of gadget's usage and (vi) trauma due to various forms of violence and abuse, including domestic violence, divorce, sexual harassment and violence, or bullying); (C) issues of adolescence (comprising dimensions of (vii) dealing with peer-pressure and intimidation, (viii) bullying in school, (ix) identity’s searching, (x) self-confidence and resilience, as well as (xi) tendencies of cutting/self-harming or suicidal behavior); (D) issues of adulthood (encompassing dimensions of (xii) depression, stress, anxiety and sorrow, (xiii) quarter-life crisis, (xiv) suicidal attempt, (xv) ineffective/inappropriate/aggressive parenting, (xvi) occupational stress, (xvii) burden of the sandwich generation, (xviii) workplace/hierarchy intimidation and (xix) behavioral maturity); (E) stigma and discrimination (against (xx) individuals with mental health disorders); (F) issues of elderly (focusing on dimensions of (xxi) dementia and (xxii) Alzheimer); (G) access to mental health assistance (covering dimensions of (xxiii) detection (screening & diagnosis), (xxiv) inadequate support and (xxv) self-diagnose); (H) lack of governmental support in managing mental health issues (due to (xxvi) lack of sufficient regulation/law); (I) inadequate education and information (due to (xxvii) inaccuracy of information accessed through social media and influencers).

### 3.4 Multi-pronged Recommendations

Finally, the consensus proposed five key recommen- dations to improve mental health awareness in Indonesia. The first recommendation stated a necessity to develop mental health-related campaigns and movements with an emphasis on specific issues in Indonesia. The personalized approach could contribute to improving the current awareness of mental health in Indonesia. An example of a successful mental health campaign/program from other countries that could be beneficial and can be adapted for Indonesia is Mental Health First Aid in Australia that are effective in improving mental health literacy.

The second recommendation stressed the importance of conducting combined clinical-community research to justify and substantiate the magnitude of various mental health issues and the impacts of the proposed solutions. An integrated approach would help to advocate for increased funding for mental health treatment, sufficient training for public health workers, and enhanced enforcement of legislation related to mental health issues.

The third recommendation emphasized the incor- poration of mental health awareness within the teaching curriculum at the level of elementary education level and the awareness of mental health issues among family members. This initiative aimed to reduce stigma and discrimination as well as equip individuals with sufficient knowledge and skills to appropriately address mental health issues.

The fourth recommendation stated a necessity to develop and further improve telemedicine technology and app-based innovation for screening and diagnosing mental health issues. By leveraging the technology, accessible and user-friendly applications could be developed to enhance awareness and knowledge of mental health among the public and skilled healthcare workers.

The fifth recommendation emphasized the importance of having innovative mental health first aid with wide access to all populations. This indicated a need to develop a system for implementing mental health first aid across various regions of Indonesia, to ensure accessibility to effective mental health treatment and to reduce stigmatization of mental health patients and inadequate management of mental health issues.

## 4 DISCUSSION

Fostering well-being and preventing mental illness necessitates efforts at both the individual and societal levels. While mental health is experienced personally, it also impacts families and can lead to broader economic and social repercussions. Addressing mental health on a population level involves tackling structural factors like poverty, violence, and inequality. Consequently, govern- ment policies, legislation, and social organizations must prioritize both the prevention of mental illness and the enhancement of overall well-being. This approach includes various strategies, from direct individual support to comprehensive population-based policies guided by the socio-ecological framework. For example, social security policies can influence mental health by addressing income inequality and affecting personal income and employment, which in turn can impact mental well-being [30–32].

The effectiveness of population-based policies across developed nations is varied. Through learnings from the United States of America, Purtle *et al*. highlighted that addressing mental health issues has largely focused on providing clinical services to individuals, rather than fostering conditions that support mental health, promote well-being, or prevent mental illness [33]. While clinical services greatly benefit many individuals and their families, relying solely on these services is not the most effective strategy for improving mental health on a population-wide scale. Despite enthusiasm for population-based mental health approaches, there is a lack of clear guidance and integrated evidence of their effectiveness. Population-based interventions should be nonclinical and exclude direct mental health services, such as psycho- therapy and medication, for individual patients. This requirement aligns with the traditional definition of population-based health approaches and the criteria set by the Public Health Accreditation Board of the United States of America for activities deemed population-based in the accreditation of public health departments. These approaches are divided into three main areas: (a) policy interventions by legislators and public agencies, (b) public health practice interventions by health department officials, and (c) healthcare system interventions by hospital and system leaders. Future strategies for advancing population mental health should focus on addressing emerging risks and seizing new opportunities. Key risks include mitigating the mental health effects of climate change and understanding the impacts of harmful social media use, such as cyberbullying, especially among young people. Technological advancements, like smart- phones, present opportunities to identify individuals in distress, connect them to mobile interventions, and support care management. For broader adoption of population-based mental health approaches, a societal shift is necessary from treating mental health as an individual issue handled solely by professionals to viewing it as a public health concern that requires input from various sectors. Institutionalizing these approaches may involve structural changes in financing, training, and accreditation. More research is needed to evaluate the effectiveness of these strategies and ensure they reduce mental health disparities. Although additional evidence is required, existing knowledge supports specific actions to improve population's mental health [33].

Other examples of population-based policies come from Australia and the United Kingdom. The term “mental health literacy” was first coined to describe “knowledge and beliefs about mental disorders that help in their recognition, management, or prevention.” Unlike other programs from that time focused on reducing stigma and discrimination, this concept represents a broader, more positively oriented public mental health objective. Nevertheless, mental health literacy still acknowledges stigma as a significant public health issue. The latest definition of mental health literacy explicitly includes reducing stigma as a key element [34,35]. The Mental Health First Aid training in Australia, aimed at improving mental health literacy in specific professional groups or population subgroups, has shown positive results [36]. This result needs to be cautiously interpreted, however, because the training only reached 1% of the Australian population. Next, the evaluation of England's first mental health literacy program, Every Mind Matters, revealed challenges in recognizing signs and symptoms of common mental disorders due to their normalization. Launched in October 2019 by Public Health England, the program aimed to encourage positive mental health actions and reduce common mental disorders through a social media campaign promoting digital resources on sleep, stress, anxiety, and low mood. The campaign initially targeted adults before the first national COVID-19 lockdown and was adapted during the pandemic to address emerging mental health challenges. Feedback led to revisions focusing more on actionable advice rather than just recognizing symptoms. The program faced challenges in providing inclusive, accessible information while avoiding fear-based avoidance, considering social issues like economic inequality, and preventing over-medicalization of mental health [37].

The population-based policies in improving mental health require continuous improvement. This is clearly shown from a recent umbrella review, in which Shah *et al* utilized a systematic review methodology to assess which national or population-level interventions or policies that address the social determinants of mental health have evidence of an effect on mental health and wellbeing [38]. Five relevant databases were investigated between January 1st, 2000, and July 23rd, 2019 to identify systematic reviews of population-level interventions or policies addressing a recognized social determinant of mental health and collected mental health outcomes. There were no restrictions on country, sub-population, or age. Twenty studies were reviewed with a note that most of them were of low or critically low quality. The primary studies were predominantly observational and conducted in higher-income settings. Higher-quality evidence suggests that more generous welfare benefits might reduce socioeconomic disparities in mental health outcomes. Lower-quality evidence indicates that inter- ventions such as unemployment insurance, improved housing, neighbourhood renewal, paid parental leave, gender equality policies, community-based parenting programs, and less restrictive migration policies could enhance mental health outcomes. Additionally, low-quality evidence suggests that limiting access to lethal means and implementing multi-component suicide prevention programs may lower suicide risk. Overall this umbrella review found that the evidence for national-level interventions addressing social determinants of mental health is limited and generally of low quality. The strongest evidence supports welfare and employment support, which should be prioritized by policymakers. Other interventions recommended for consideration include gender equity policies, less restrictive migration policies, restricting access to means of suicide, multi-component suicide prevention campaigns, and broad community parenting programs. The review highlights a need for higher-quality primary research and better reporting on the effects of conditional benefits, neighboruhood renewal, and mixed-income housing. No systematic review evidence was found for national strategies related to violence reduction, environmental hazards, community services, or age and ethnicity-inclusive policies. While there is a broader evidence base for locally evaluated interventions, these need further evaluation to determine their effectiveness at the national level [38].

Taken together, current evidence supports the importance for Indonesia to have its own national or population-based policy to improve mental health awareness in Indonesia. Despite the upcoming challenges to effectively implementing Indonesian population-based intervention, we hope that our five recommendations could increase mental health awareness, reduce stigmatization of mental health patients and inadequate management of mental health issues, as well as could ensure accessibility to effective mental health treatment in Indonesia.

## CONCLUSION

Mental health is a fundamental human right, which sustains individuals’ abilities to connect, function, cope and thrive in multiple aspects of life. Disruptions in mental health would disturb human well-being and would create economic consequences as well. As one of the low and middle-income countries, Indonesia will bear substantial impacts of mental health conditions if it does not start to prioritize mental health awareness and provide sufficient resources to screen, diagnose, and treat individuals with mental health disorders. By focusing on four high-risk population groups, the expert consensus identified nine specific themes of mental health issues in Indonesia and subsequently proposed multi-pronged recommendations to start improving mental health awareness in Indonesia.

## Figures and Tables

**Fig. (1):**
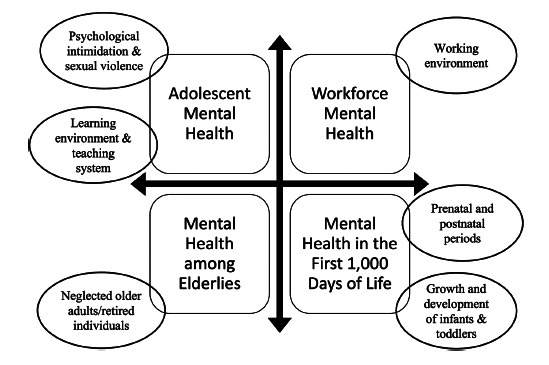
Six major mental health priorities in Indonesia across four high-risk population groups.

**Table 1:** Identified specific mental health issues in Indonesia that cover 9 broad themes and 28 dimensions.

**Theme**	**Dimension**
(A) Issues of pregnancy and early parenting	(i) Prenatal stress (ii) Baby blues syndrome
(B) Issues of childhood 1.	(iii) Neurodevelopmental disorders (iv) Emotional and behavioural problems (v) Impact of gadget usage (vi)Ttrauma due to various forms of violence and abuse, *e.g*., domestic violence, divorce, sexual harassment and violence, or bullying
(C) Issues of adolescence	(vii) Dealing with peer pressure and intimidation (viii) Bullying in school (ix) Identity’s searching (x) Self-confidence and resilience (xi) tendencies of cutting/self-harming and suicidal behaviour
(D) Issues of adulthood	(xii) Depression, stress, anxiety and sorrow (xiii) Quarter-life crisis (xiv) Suicidal attempt (xv) Ineffective/inappropriate/aggressive parenting (xvi) Occupational stress (xvii) Burden of the sandwich generation (xviii) Workplace/hierarchy intimidation (xix) Behavioural maturity
(E) Stigma and discrimination	(xx) Individuals with mental health disorders
(F) Issues of elderly	(xxi) Dementia (xxii) Alzheimer
(G) Access to mental health assistance	(xxiii) Detection (screening and diagnosis) (xxiv) Inadequate support (xxv) Self-diagnose
(H) lack of governmental support in managing mental health issues	(xxvi) Lack of sufficient regulation/law
(I) Inadequate education and information	(xxvii) Inaccuracy of information accessed through social media and influencers

## Data Availability

The data and supportive information is available within the article.

## LIST OF ABBREVIATIONS

SDGs: = Sustainable Development Goals
WHO: = World Health Organization
YLLs: = Years of Life Lost
